# Editorial: Cell-based neurodegenerative disease modeling

**DOI:** 10.3389/fcell.2023.1323954

**Published:** 2023-10-26

**Authors:** Yohan Oh

**Affiliations:** ^1^ Department of Biomedical Science, Graduate School of Biomedical Science and Engineering, Hanyang University, Seoul, Republic of Korea; ^2^ Department of Biochemistry and Molecular Biology, College of Medicine, Hanyang University, Seoul, Republic of Korea; ^3^ Hanyang Institute of Bioscience and Biotechnology, Hanyang University, Seoul, Republic of Korea; ^4^ Hanyang Institute of Advanced BioConvergence, Hanyang University, Seoul, Republic of Korea

**Keywords:** neurodegenerative disease, disease modeling, human pluripotent stem cell, cell therapy, Parkinson’s diseae, amyotrophic lateral sclerosis

Age-related neurodegenerative diseases, including Alzheimer’s disease (AD), Parkinson’s disease (PD), and amyotrophic lateral sclerosis (ALS), continue to be formidable challenges to the global medical community. As the global population ages, the prevalence of these diseases is also expected to increase. One of the most significant challenges in the fight against these diseases is the lack of effective cell-based models that can accurately recapitulate the complex pathophysiology observed in human patients. However, with recent advancements in stem cell technology, particularly human pluripotent stem cells (hPSCs), there is a beacon of hope.

This Research Topic, titled “Cell-Based Neurodegenerative Disease Modeling,” brings together an array of studies that showcases the current strides in developing, improving, and validating cell-based neurodegenerative disease models. This is a collation of pioneering research articles and reviews from experts in the field, providing insights, methodologies, and challenges pertinent to the cellular models of these debilitating diseases.

In a comprehensive review, Lin et al. have investigated the potential of cellular therapy for ALS, a disease with unclear pathology and ineffective therapeutic modalities. They emphasized the immunomodulatory effects and potential protective measures of cellular therapy for motor neuron circuits in ALS while highlighting the ongoing debates on the safety and efficacy of such interventions.

Further advancing our understanding of PD, a Lewy body disease, Natalwala et al. have reported the development of an isogenic Research Topic method of pluripotent stem cell lines with elevated α-synuclein expression. Their findings reinforce the capability of pluripotent stem cell lines, even with increased α-synuclein levels, to efficiently differentiate into dopaminergic and cortical neurons. Such models have become invaluable tools for studying PD-associated early molecular changes.

Sibuea et al. have studied the complex territory of maturation in stem cell-derived dopaminergic neuron. They have meticulously investigated the role of culture additives commonly used in maturation, like dbcAMP and TGFβ3, shedding light on their nuanced impact on the phenotype of midbrain dopaminergic neurons, thereby offering a fresh perspective on the current protocols.

Drawing attention to the critical role of endoplasmic reticulum–mitochondrial contact sites (ERMCS) in neurodegenerative diseases, Yokota et al. have employed tyrosine hydroxylase reporter iPSC lines to elucidate the aberrations in ERMCS and mitochondrial Ca^2+^ flux in PRKN-mutant patient dopaminergic neurons. These findings emphasize the significance of studying the ERMCS to enhance our understanding of dopaminergic neuronal degeneration in specific patient populations.

Moon et al. have holistically reviewed the intricate challenges associated with cell therapy for PD using hPSCs. By focusing on *in vivo* differentiation protocols and transplantation results, they underscored the potential of hPSC-derived ventral midbrain dopaminergic progenitors for PD treatment, while simultaneously focusing on current hurdles, such as lineage identification.

This Research Topic of articles emphasizes the critical importance and urgency of refining and developing cell-based models, particularly those based on hPSCs, to emulate human neurodegenerative diseases. These models, as shown in this Research Topic, not only provide invaluable insights into disease pathogenesis, but also present an avenue for testing potential therapeutic interventions.

In conclusion, as the gap between traditional animal and human cell models continues to widen, pioneering studies such as those presented here have become vital. I hope that this compilation will serve as a touchstone for future endeavors in neurodegenerative disease modeling and therapeutics.

